# A Clinical Perspective on the Role of Electronic Devices in Monitoring and Promoting Adherence in Airways Disease

**DOI:** 10.3389/fmedt.2021.604475

**Published:** 2021-04-12

**Authors:** Vincent Brennan, Christopher Mulvey, Garrett Greene, Elaine Mac Hale, Richard W. Costello

**Affiliations:** ^1^Clinical Research Center, Royal College of Surgeons in Ireland, Dublin, Ireland; ^2^Beaumont Hospital, Dublin, Ireland

**Keywords:** asthma, COPD, adherence, personalized, objective

## Abstract

Poor adherence to treatment is a common reason why patients with chronic disease have worse outcomes than might be expected. Poor treatment adherence is of particular concern among people with airways disease because, apart from not taking treatment as prescribed, inhaled medication can also be administered incorrectly. Recently, a number of technological advances that accurately document when an inhaled treatment has been used and, in certain instances, how it was used have been developed. There is good evidence from a number of research groups that these devices, either by patient reminders or physician feedback, promote adherence to inhaled treatments. What is less certain is how, in a real-world setting, these devices change outcomes. In this perspective article, the role of electronic devices in quantifying treatment use and addressing poor treatment adherence and their potential role in clinical practice outside of clinical validation trials are described.

## Introduction

It is purported that the physician of ancient times, Hippocrates, stated that “[the physician] should keep aware that patients often lie when they state that they have taken certain medications.” So important is the issue of medication adherence that even the World Health Organization has addressed this challenging problem. In a seminal publication from 2003, it was reported that, in the developed world, adherence to prescribed treatments was believed to be as low as 50% (1). Since that landmark report, there has been little in the way of published research to dispute this.

There are many consequences of poor adherence. Arguably, the most significant of them is that poor adherence is not recognized by the patient's physician. Partially treated conditions, arising from poor adherence, inevitably lead to the prescription of additional medications or unnecessary investigations. This results in increased downstream healthcare costs through worsening of chronic conditions, as well as the costs and side effects of more advanced treatments. Working out the cost of poor treatment adherence is challenging as it is difficult to distinguish an uncontrolled condition from one that simply did not receive enough treatment. This means that there is a large variation in estimated values; nonetheless, the cost is substantive. A recent systematic review suggests that the cost of inadequate treatment adherence is between $100 billion and $290 billion per year in the United States with between $949 and $44,190 per person per year among people with respiratory diseases (2015 US dollars) (2).

Poor treatment adherence to inhaled therapy appears to be a particularly pervasive problem, as demonstrated by Krigsman et al. (3) who compared adherence to diabetic medication with inhaled medications in patients diagnosed with both diabetes and asthma or chronic obstructive pulmonary disease (COPD). While 68% of patients demonstrated satisfactory primary adherence for diabetes medication, only 42% of the same cohort had a satisfactory primary adherence rates for their inhaled medication.

Clearly, poor treatment adherence is a major public health problem. To better understand treatment adherence, it is necessary to obtain objective data as self-reported data are not accurate (4). Electronic patient health records and pharmacy dispensing records can provide a means for assessing if an individual initiates a treatment and can also determine the duration a patient persists with that treatment. Sensor-enabled electronic devices go beyond this and can be used to determine when, and if, the treatment was used. This is a clinical perspective on the role of electronic devices in monitoring and promoting adherence as well as how this information can be used to better understand and address causes of poor treatment adherence.

## Causes of Poor Adherence to Inhaled Treatment

The root causes of poor adherence to inhaled therapy are multifactorial (5), and as a result, the quoted statistic of 50% adherence perhaps oversimplifies the issue. Gender, ethnicity, and socioeconomic status are among the factors associated with poor treatment adherence in respiratory conditions (6). Within these broad groupings, issues such as access to and affordability of medicines, broadly termed barriers, are factors that drive adherence. Other factors that reflect the social and economic dimensions of health and healthcare, such as how healthcare is delivered and how clinicians communicate with their individual patients, influence treatment adherence. For most people, in whom barriers to healthcare are not the primary cause of poor adherence, elegant work from Horne et al. has shown that people weigh up the perceived necessity of the proposed treatment with their own personal concerns about the treatment and the underlying condition (7).

As treatment adherence is determined by personalized behavioral and sociological factors, it is not surprising that adherence to treatment is not determined by disease severity. In a landmark study, Gamble et al. (8) reported that only 21% of patients in a specialized severe asthma clinic collected their inhaled medication as prescribed, and 35% of patients collected fewer than half of their prescriptions for inhaled corticosteroids (the mainstay of asthma treatment). Similar results were described by Sulaiman et al. (9) in patients with COPD, where regular and correctly taken preventer treatment was seen in only 6% of patients in the month following hospital care. These data indicate the potential scale of the problem for conditions such as asthma and COPD.

**Figure 1:**
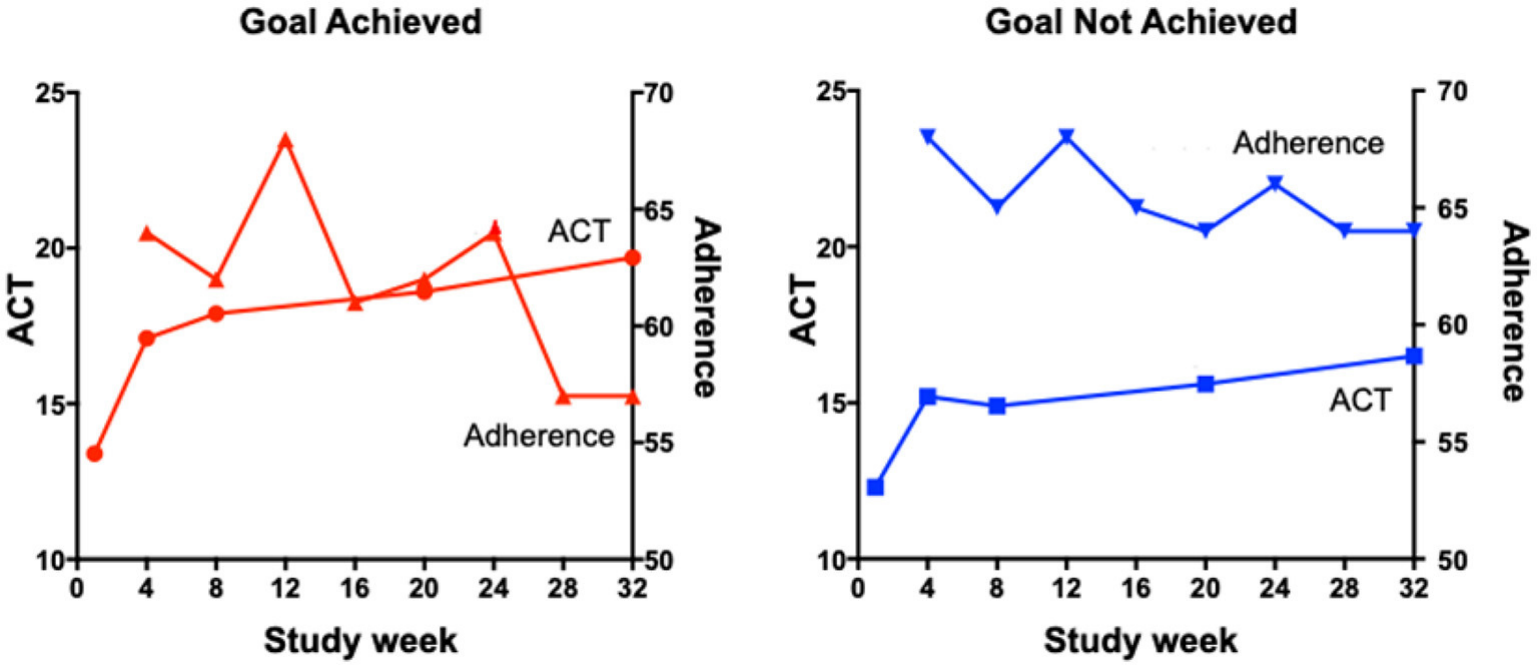
The relationship between ACT scores and adherence in participants who did or did not achieve their goals.

There are a number of proposed reasons for poor treatment adherence that are particularly relevant or specific to inhaled therapy. First, many preparations of inhaled therapy for chronic airways diseases require twice-daily dosing and higher-frequency dosing is associated with reduced adherence (10). A small study from 2019 comparing the effectiveness of a once-daily preparation of preventer medication to a standard twice-daily regimen noted a statistically significant reduction in forgetfulness and inconvenience as measured by a questionnaire (11). Second, asthma symptoms are known to vary with the time of day, the season, and even where the person lives and works, as seen in occupational and allergic asthma. These fluctuations in symptoms can make it difficult for patients to see a relationship between treatment adherence and clinical symptoms. Recently, Mulvey et al. (12) have shown that once a patient achieves their personal asthma treatment goal, there is a subsequent decline in adherence (Figure 1). These data indicate how person-specific behaviors can influence treatment adherence.

Errors in inhaler technique are common and result in worse control, which in turn leads to increased use of healthcare including hospital admission and rescue oral corticosteroids (13, 14). In a large systematic review, it was determined that nearly 70% of patients demonstrated a suboptimal inhaler technique (15), and a smaller study looking at the same subject showed that only 12% of people were able to use their inhaler correctly. Even though the vast majority of patients showed poor inhaler technique during the study, all participants reported feeling confident using their devices (16). This highlights the need for ongoing surveillance of inhaler technique (17). The most efficient method for correcting poor inhaler technique is unclear (18), and while face-to-face verbal instruction appears to be more effective than other methods (18, 19), the results are rarely sustained (20, 21). These findings have prompted investigators to develop a number of devices that can accurately and objectively assess inhaler use.

## Electronic Monitoring Devices Used With Inhalers

In an attempt to further understand when and how treatment is used, so called implementation adherence, a number of electronic monitoring devices have been developed for use with inhaled treatments. The first device was the Nebulizer Chronolog (22). This innovative device provided investigators a simple digital readout that detailed the number of inhalations and time that these were taken. The first study with this device, published 35 years ago, described poor adherence to a novel treatment in a clinical trial. Both poor concordance with self-report and the potential effects of insufficient treatment use in a clinical trial were described. Subsequently, a number of similar devices were designed (23), but this first wave of electronic monitoring devices had limited use in real-world practice. It is not clear why these were not adapted into practice.

With the advent of low-cost sensors, Bluetooth data transmission, cloud storage, and mobile web applications, a new generation of devices has been developed and validated (Table 1). Adherium (Auckland, New Zealand) designed a number of add-on devices, collectively termed the “Hailie Solution.” The device is a plastic casing that contains a battery and clock that timestamps each actuation of the inhaler. These data are communicated to an application on the patient's smartphone by Bluetooth, allowing the patients and their physician to review their treatment use over a period of time and identify which doses are being missed. The Hailie solution also sends a reminder to the smartphone when a dose is due. Propeller Health (Res Med, Wisconsin, USA) also created an externally attached device designed to work with a wide variety of devices. Like the Hailie solution, the Propeller devices are designed to work with a smartphone application giving the patient and physician an accurate record of treatment adherence as well as providing reminders to the patient. Additionally, the Propeller device has GPS (global positioning system) capabilities, and so it records not only the time the device is actuated but also the location. This, in combination with a symptom diary, may allow the patient to identify areas or activities that may be asthmogenic, enabling them to avoid certain triggers. The Propeller device also has a public health use as patients are required to consent to disclosure of their records to public health agencies, enabling the early identification of significant pollution events, which may be detrimental to patients with chronic airways diseases.

**Table 1:** Bolt-on and smart devices.

**Bolt-on devices with FDA approval**
Propeller (24)
Adherium—Hailie (formerly SmartInhaler) (25)
**Bolt-on devices without FDA approval**
Findair (26)
AptarPharma—Adhero (formerly Cohero) (27)
Aerobit Health
Capmedic (28 )
Breathesuite MDI (29)
INCA (30)
**Smart devices with FDA approval**
Teva Digihaler (31)
**Smart devices without FDA approval**
H&T Presspart—eMDI (32)
Kindeva—the intelligent control inhaler (in development)
Nypro—Ruby (not yet in market)

Continuous patient feedback combined with easy-to-use smartphone applications addresses one cause of poor adherence, namely, that patients are prone to forget their treatment or not bother to use it when they feel well or are not symptomatic. These devices also allow the patient to develop a better understanding of their disease and may help counter some fixed beliefs about the treatment efficacy. There is good evidence of effectiveness of these devices in clinical trials in a variety of clinical scenarios, mostly among patients attending primary care (24, 33–38). These devices do not objectively record inhaler technique, limiting their use beyond merely accurately recording when the treatment was used.

Recently, electronic devices that are integrated within the inhaler have been launched commercially. One such device, the Digihaler (39), is preloaded with albuterol (ProAir), fluticasone propionate (ArmonAir), or fluticasone propionate and salmeterol (AirDuo), and all have received US Food and Drug Administration (FDA) approval for use in asthma. The device contains Bluetooth wireless technology and sensors that can accurately measure inhalation parameters including peak inspiratory flow rate in real time. This allows for the patient, as well as the physician, to review not only their time-stamped adherence but also the efficiency of their drug delivery.

The first device to also assess time and technique of use was the INCA device (30). This, like the Adherium and Propeller devices, is a “bolt on” device used with a diskus device. It contains a microphone and a microprocessor (Vitalograph Ltd, Ennis, Ireland) creating an audio recording of each inhaler use. This recording is analyzed by software that can identify errors in inhaler technique including low peak inspiratory flow rate, exhaling into the mouthpiece and dose dumping—when the inhaler is primed and loaded, but the drug is not inhaled. The INCA device gives the physician a time-stamped adherence and technique report allowing for targeted and personalized education (40–42).

## The Clinical Impact of Using Electronic Monitoring Devices With Inhaled Medication

A number of investigators have used electronic monitoring devices as a tool to improve adherence; these important randomized controlled trials (RCTs) are summarized in Table 2. Four of the eight RCTs had adherence as their primary endpoint, and the others listed adherence as a secondary endpoint. Adherence rates were variable, ranging from 30 to 84%. Two trials delivered adherence-promoting advice by healthcare worker feedback alone, two had automated reminder prompts alone, and four had a combination of healthcare worker feedback and prompts. The trials all reported significantly increased rates of adherence when the electronic monitoring devices were used compared with usual care.

**Table 2:** Randomized controlled trials evaluating the effect of electronic monitoring devices on disease control.

**Authors**	**Aim**	**N (randomized) and arm description**	**Primary endpoint(s)**	**Secondary endpoints of note**	**Results and comments**
Chan et al. (25)	To evaluate if use of an audiovisual reminder improves asthma management in children	220 (110 standard care, 110 audiovisual reminder)	• Adherence to preventive inhaled corticosteroids • Number of school days missed (for any reason)	• Change in childhood asthma control test (ACT) score • Change in asthma morbidity score • Changes in FEV_1_ • No. of ED attendances • Exacerbation rates • Caregiver absence from work • Number of days with reliever use	• Median adherence rate of 84% in the active group and 30% in the control group (*p* < 0.0001). • There was no difference in days off school for any reason between the groups. • Both groups had improvements in asthma morbidity score and childhood ACT, although the degree of improvement was higher in the intervention group. • No significant difference in FEV_1_ between groups. • The intervention group had significantly fewer parent-reported asthma exacerbations in the first 2 months (6 vs. 26%), but this was not seen in the subsequent two time points. • No significant between-group difference in ED visits or caregiver days off. • Reliever use was significantly less in the intervention group (9.5% of days vs. 17.4%, *p* = 0.002).
Morton et al. (37)	To evaluate if electronic adherence monitoring with feedback and alarms improves clinical outcomes in children with poorly controlled asthma	90 (47 reminder and personalized feedback at clinic, 43 usual care)	Change in Asthma control questionnaire (ACQ) score	• Adherence rates change in FEV_1_ • No. of unplanned medical reviews for asthma (ED or GP) • No. of courses of oral steroids • No. of days off school due to asthma • Reliever use • miniPAQLQ	• Both groups had a decrease in their ACQ score at 3 months (1 in control group, 0.9 in active group). This was maintained throughout the study, and there was no significant between-group difference. • Adherence was higher in the intervention group at 70 vs. 49% (*p* < 0.001). • No difference in ED/GP attendances • No difference in days off school. • There was no between-group difference for reliever use or miniPAQLQ. • The intervention group received fewer courses of oral corticosteroids and had fewer hospital admissions. • There was no significant difference in FEV_1_.
Foster et al. (38)	To evaluate the effectiveness of two brief GP delivered interventions for improving adherence and asthma control vs. standard care	143 (43 usual care, 24 personalized adherence discussion, 35 inhaler reminders and feedback, 41 personalized adherence discussions, and inhaler reminders and feedback)	Change in ACT score	• Change in mini AQLQ/Hospital Anxiety and Depression Scale • Medication Adherence Report Scale for asthma • Change in FEV_1_ • Number of steroid courses (self-reported data compared with GP records)	• All groups had a significant improvement in their ACT, which was maintained over 6 months, 4.5 ± 4.9; *p* < 0.0001. • There was no significant between-group difference in ACT, *p* = 0.14. • Adherence was higher in patients who had reminders in comparison to those who did not (73 vs. 46%), *p* < 0.0001. • There was no significant between-group change in FEV_1_. The active group had fewer exacerbations (11%) when compared with the control group (28%) (*p* = 0.013),
					but when clustering and previous self-reported oral steroid use was accounted for, this difference did not reach statistical significance, *p* = 0.06. • There were clinically important improvements in asthma-related quality of life and anxiety scores, but there was no between-group difference.
Moore et al. (43)	To evaluate the effect of a connected inhaler system on adherence to maintenance therapy in participants with uncontrolled asthma	437; (87 Reminders and feedback on maintenance inhaler, 88 reminders only on maintenance inhaler, 88 reminders and feedback on all inhalers, 88 reminders only for all inhalers, 86 usual care)	Adherence to maintenance therapy	• Reliever medication usage • ACT score • FeNO • Peak expiratory flow	• Adherence was significantly higher in the electronic monitoring group over the latter half of the study (80.9%) n the active group compared to the 69% in the control arm, *p* < 0.001. • Use of a monitoring system on the patient's reliever device resulted in reduced total usage this was only statistically significant when these groups were compared with the control group. • There was no between-group difference in change in ACT score with >65% of patients in each arm having an ACT ≥20 or having shown a rise in ACT score of >3 points. • There was no change in FeNO or peak expiratory flow throughout the study although the authors noted a difference in these parameters at enrollment when compared to randomization.
Mosnaim et al. (44)	To evaluate the effect of patient self-monitoring with a smartphone enabled electronic medication monitoring system plus remote clinician feedback influences inhaled corticosteroid (ICS) and short acting beta-2 agonist (SABA) use	100 (75 reminders via smartphone app, 25 usual care)	The difference in SABA-free days from run-in to the last 2 weeks of the study	• Adherence rates between groups during the final 2 weeks vs. the run-in period • Change in SABA-free days • Change in ACT >3 points • Exacerbation rates	• The control group adherence decreased by 15% over the study compared to 2% in the active group, *p* < 0.01. • There was a significant rise in the percentage of SABA free days in the control group from 58 to 77% (*p* < 0.01). • There was also a rise in SABA free days in the control group from 75 to 81%, but this did not reach significance (*p* = 0.18). • Asthma control and percentage of patients with exacerbations did not differ between the groups over the study period.
Merchant et al. (24)	To evaluate the real-world effectiveness of the Propeller Health Asthma Platform in reducing SABA use and improving asthma control.	495 (250 reminders and physician feedback, 245 usual care)	The relative reduction in SABA use	• Change in proportion of patients with controlled asthma based on ACT >19 • Change in ACT	• Both groups reduced SABA usage over the study, but the intervention group had a significantly larger reduction in SABA uses/person per day, *p* < 0.001. This was also seen in the percentage of SABA-free days, *p* < 0.01.
					• Both groups demonstrated an increase in ACT and proportion of patients with ACT >19, but there were no significant between-group differences. • Subgroup analysis of patients who were originally uncontrolled demonstrated a statistically significant rise in ACT and proportion of patients with ACT>19 in the intervention group compared with the control group, *p* < 0.01 and < 0.05, respectively.
O'Dwyer et al. (45)	To evaluate the effect on inhaler technique and adherence when patients receive personalized feedback informed by an electronic monitoring device vs. standard care or inhaler technique demonstration	152 (74 healthcare professional feedback, 56 to demonstration arm, 22 to usual care)	The rate of actual adherence (actual adherence refers to attempted adherence minus the times when there was a critical inhaler technique error)	• Attempted adherence rate • Inhaler technique • Proportion of patients to adherence rates >80 or < 50% • Change in St. George Respiratory Questionnaire • Patient reported symptoms (patient recorded diary) • Exacerbation rate	• Adherence rates in the biofeedback group were better (62%) than the demonstration group (44%) and the control group (38%) at month 2. • At month 6, the biofeedback group had adherence rates 31% higher than the control group (*p* = 0.01), but the difference between the biofeedback and demonstration group was not statistically significant at 7%. • There were significantly more patients with adherence more than 80% and significantly less with adherence < 50% in the biofeedback group compared with other groups, *p* < 0.05. • Both intervention groups had a reduction in SGRQ scores, but only the biofeedback group maintained a statistically significant reduction at 6 months, *p* = 0.04. • There was a significant reduction in all asthma symptoms in the biofeedback group. • There were no significant between-group differences in the exacerbation rates.
Sulaiman et al. (46)	To evaluate the effect of visual (bio)feedback to the patient on their specific components of adherence on adherence rates	218 (111 biofeedback from healthcare professional, 107 intensive education arm, usual care)	• The difference in the rate of “actual adherence rate” between groups • Cumulative drug exposure for the last month of the study	The proportion of patients with truly refractory asthma vs. those with uncontrolled asthma and poor treatment adherence	• The rate of actual adherence during the third month in the biofeedback group was 73% as opposed to 63% in the intensive education group, *p ≤* 0.01. • At the end of the study, 63% of the study participants remained uncontrolled, but only 27% would be considered refractory as their adherence was <80%.

Some aspects of disease control, such as reliever use and hospital admissions, were shown to be improved when the electronic monitoring devices were used to promote adherence. Six of the eight RCTs among asthma patients assessed reliever use or percentage of reliever-free days as endpoints of their studies. Five of these showed a significant reduction in reliever use when there was feedback from an electronic monitor. The study of Morton et al. (37) was the only study that did not show a significant reduction in reliever use. Similarly, results were seen in a single-arm study of patients with COPD in whom there was a 49% reduction in “reliever” use and a 91% increase in symptom-free days after a period of 6 months (34). Exacerbation rates were a secondary outcome in five studies. The definition of an exacerbation was variable, and the results were mixed; for example, Morton et al. noted fewer courses of prescribed oral steroids and fewer hospital admissions with the use of an electronic monitoring device, but there was no between-group difference in emergency department (ED) attendances or unscheduled general practitioner (GP) visits. Foster et al. (38) originally found a statistically significant difference in exacerbation rates, but when more detailed analysis looking at patients preceding behavior was performed, this effect was no longer seen.

Compared with adherence, self-reported disease control was less clearly affected. Asthma control questionnaires were the primary endpoints in two of the above RCTs and secondary endpoints in the other six. While there was frequently a numerically better improvement in the self-reported disease control scores for the group who received a treatment adherence intervention compared to the control group, this often did not reach statistical significance. It is, however, important to note that the control groups in these studies also reported improvement in their asthma control.

Three trials reported forced expiratory volume in 1 second (FEV_1_) as an outcome, but there was no statistically significant difference between those using an electronic monitoring device compared to those without. Only one trial recorded peak expiratory flow rate as a secondary endpoint that did not significantly change, although there was a noted increase between screening and the start of the study. Moore et al. (43) also measured fractional exhaled nitric oxide (FeNO) as a secondary endpoint and also noted a change between screening and the start of the study with no further change. Hence, despite the apparent improved treatment adherence from the use of electronic devices, this does not reliably confer improved clinical outcomes.

## Measurement of Adherence Using Digital Devices

The major advantage of digitally enabled inhalers over the existing manual dose counter is that they are time-stamped and therefore record when the inhaler was used, allowing for the exact pattern of inhaler use to be assessed. Original measurements of adherence were quantified as the number of days that treatment doses were taken. This was reported as the mean daily dose. This was a logical approach when drug treatments were complex (i.e., more than once a day), and the aim was to assess the impact of an adherence intervention. In this case, one could assess the proportion of time that a patient was adherent to the prescription throughout the period of observations.

This measure, however, is not satisfactory when adherence changes are linked to clinical outcomes. Although some trials have shown an improvement in objective measures such as reliever use and healthcare utilization as described above, most clinical studies with digital devices have been disappointing, showing only a modest clinical impact. This may be due to the heterogeneity of non-adherence patterns of the different pharmacodynamic effects. Take, for example, two patients, patients A and B who both have 75% medication adherence over a 28-day period. Patient A misses every fourth inhalation, whereas patient B took the inhaler correctly but missed an entire week at the end of the monitored period. In the case of patient A, the occasional missed dose will lead to a small loss in overall drug concentration over the study period, whereas patient B will have had a completely different course where simple pharmacokinetic principles show a steady decline in drug effect and a slow rise after resuming drug administration.

In an effort to relate the effects of intermittent or unintentional poor treatment adherence to clinical impacts, Sulaiman et al. (47) evaluated the inhaler technique and the effect of the time interval between medication doses. They defined a measure of adherence as the adjusted area under the curve. The “adjustment” was a weighted score to account for errors in both timing and technique (48, 49). This metric, referred to as the “actual AUC,” was more closely related to changes in lung function and quality of life than the more readily available and commonly used measures of adherence such as the mean daily dose (47). Building on this concept, Greene et al. (50) described a novel method of measuring adherence, time above threshold. The treatment threshold represents the drug's effectiveness, for example, suppressing airway inflammation or inducing maximal bronchodilation. This method is more predictive of clinical events than any other measure. Hence, when using an electronic adherence device, measures that relate drug adherence with pharmacokinetic principles may provide a better approach to understanding the relationship between adherence and clinical disease outcomes.

The adherence measurements described above reflect two clinically important issues. The mean daily dose measures how adherent an individual has been. This can be used in clinical trials to compare adherence interventions. In contrast, pharmacokinetic measures, such as time above threshold or area under the curve method, can quantify the impact of treatment and may help bridge the gap between adherence rates and clinical outcomes.

A further use of these devices, beyond personalized adherence measurement, is that they can be used to provide insight into patient health behaviors. Cushen et al. (51) evaluated adherence in patients with COPD and identified four specific clusters of adherence patterns among patients with similar physiological levels of COPD. One group used their treatment regularly and with good technique. This group had good cognition scores and good social support and had the least anxiety. In follow-up over a year, this group had the best health outcomes. A second group regularly used their treatment but had poor inhaler technique, in effect receiving much less treatment than prescribed. Similarly, this group had good cognition, good social support, and little anxiety. They also had infrequent hospital admissions but had more mild exacerbations. The overall good health outcomes in these patients support the data from most clinical trials evaluating inhaler use in COPD, which show a limited impact on hospital admissions.

In contrast to these groups, the other two groups demonstrated irregular use of their inhalers and had very poor clinical outcomes. One used their inhaler irregularly, but when they did so, it was with good technique. This group had high levels of anxiety and poor cognition and frequently presented for unscheduled care. The fourth group also demonstrated irregular inhaler use but with poor technique. This group had the least social support and had the highest mortality (51). Similar findings were reported among trial participants by Vestbo et al. (52). *Post-hoc* analysis of data from a large clinical trial of patients with advanced COPD showed that, regardless of what treatment group patients were assigned to, patients with poor adherence had the worst outcomes (52). Hence, the data provided by electronic monitoring devices, as with many other digital technologies, yield much more than just an accurate account of a patient's adherence to that inhaler.

## Conclusion

Over the last 35 years, electronic monitoring of inhaler adherence has advanced from simple devices to ones with advanced sensors that record, automatically analyze, and communicate with patients. In this time, many large, well-performed clinical studies have confirmed that these devices can be used to improve patient adherence and some outcomes. In order for these devices to be integrated in clinical practice, the clinical and economic advantages of these technologies will need to be established. The novel methods of measurement described above may be one way of assessing the clinical and economic advantages of these technologies.

## Author Contributions

CM: manuscript editing, reviewing, and literature review. GG: manuscript editing and reviewing. EH: manuscript editing and reviewing—discussion of content. RC: oversight and authorship. All authors contributed to the article and approved the submitted version.

## Conflict of Interest

RC reports grants and personal fees from Aerogen, personal fees from Boehringer Ingelheim, grants and personal fees from GSK, personal fees from Novartis, personal fees from TEVA, outside the submitted work; In addition, Dr. Costello has a patent EP12182189.6 licensed. The remaining authors declare that the research was conducted in the absence of any commercial or financial relationships that could be construed as a potential conflict of interest.
